# How opinion variation among in-groups can skew perceptions of ideological polarization

**DOI:** 10.1093/pnasnexus/pgaf184

**Published:** 2025-06-06

**Authors:** Peter Steiglechner, Paul E Smaldino, Agostino Merico

**Affiliations:** Global Change Impacts and Adaptation, Leibniz Centre for Tropical Marine Research (ZMT), 28359 Bremen, Germany; School of Science, Constructor University, 28759 Bremen, Germany; Collective Minds, Complexity Science Hub, 1030 Vienna, Austria; Department of Cognitive and Information Sciences, University of California, Merced, CA 95343, USA; Santa Fe Institute, Santa Fe, NM 87501,USA; Global Change Impacts and Adaptation, Leibniz Centre for Tropical Marine Research (ZMT), 28359 Bremen, Germany; School of Science, Constructor University, 28759 Bremen, Germany

**Keywords:** polarization, perception, social identity, belief dynamics, climate change

## Abstract

There is a widespread perception that society has been polarizing into groups with increasingly divergent opinions. Multiple studies have sought to quantify the degree of opinion divergence (or ideological polarization), typically relying on differences between self-reported opinions, and have reached mixed conclusions. We propose this inconsistency can be explained by the way individuals’ subjective perceptions are shaped by their social identities. We introduce a formal framework to analyze opinion data that accounts for such asymmetric, dynamic perceptions. When members of an in-group become increasingly homogeneous on a given topic (i.e. when the variance of opinions in that group decreases), they perceive deviant opinions as increasingly distant from their own. Consequently, these individuals may perceive greater polarization than an objective, neutral observer would. Applying the framework to data on the opinions of Germans about climate change, we show that perceived polarization may depend as much on the dynamics of in-group variance as it does on actual opinion divergence in society. Moreover, we show that the direction of this effect may vary over time and across different partisan groups. Our framework offers an explanation why people might sometimes perceive higher levels of ideological polarization than surveys indicate, independent of social segregation, polarization-enforcing cognitive biases, or affect-driven attitudes towards out-groups.

Significance Statement Are opinions on political topics like climate change truly diverging? This is a critical question with implications for social cohesion and collective action. Empirical research suggests that people with a strong partisan political identity often perceive greater polarization than politically neutral or centrist individuals, a discrepancy that is not well understood. Our study provides a possible explanation, based on the way individuals’ perceptions of others’ opinions adapt to the distribution of opinions among their in-group. Using German opinion data about climate change, we show how the dynamics of perception can lead individuals to either overestimate or underestimate the true ideological divergence in their society.

## Introduction

Polarization is often deemed one of the most concerning social phenomena in modern times (1–3). How do we know how polarized people really are on issues of public concern, such as climate change, migration, or tax policies? Most metrics of ideological polarization are based on aggregate differences in opinion (e.g. between partisan groups) (4–7). For example, many studies measure ideological polarization on climate change in the United States as the difference in average concern expressed by Democrats vs. Republicans (8, 9). This approach is based on the assumption that differences between survey options do not meaningfully differ between populations (10). Yet, psychological evidence from multiple domains indicates that differences are typically perceived in relation to a person’s environment (11–16). What someone in a low-variance environment perceives as a large difference may be perceived as a small difference by someone in a high-variance environment. Perceptions adapt once the environment changes. This is a common effect in visual perception: in the dim light of dawn or dusk (a low-variance environment), humans can differentiate shapes that are not perfectly defined, but when the headlights of a car are turned on (changing the scene into a high-variance environment of sharp contrasts), the sensitivity to faint contrasts is reduced (17, 18). We suggest that this principle may operate similarly on the perception of differences between opinions.

Our study is based on the principle that (i) people rely on internalized belief systems to perceive the opinions of others, mapping them onto a (multidimensional) opinion landscape and that (ii) this mapping is shaped subjectively and dynamically by social contexts. For example, people in many societies tend to collapse opinions on diverse topics like climate change, immigration, or public health onto a primary ideological axis, classifying individuals as if they occupied a single location on a spectrum between “left” and “right” (19) or liberal and conservative (20). The utility and adoption of this opinion compression have increased since the mid-20th century, as left–right political belief systems became more established and coherent (21, 22). When people perceive opinions as “positions” in mentally constructed spatial representations, as the literature above suggests, it seems natural to assume that they also evaluate the opinion distance between themselves and others as a distance measured in this space—for example, they evaluate an individuals’ set of opinions as “much more left” or “slightly more right” compared to their own.

The mental representations depend on social contexts and may thus vary within a society, across societies and over time (23–26). Specifically, perception is tied to the degree of variation an individual observes (12, 16). However, people identify and compare themselves primarily with people who are similar to themselves and whom they value highly (27, 28), and the knowledge of opinions within their in-group may update faster than the knowledge of how opinions are distributed in the wider world. The opinion variation an individual expects on a specific issue may thus reflect more the variation within in-groups rather than the variation within the whole population. If this is true, then perceptions of opinion distances are subjective and many measures are likely to misrepresent the polarization that individuals see, both in terms of magnitude and in the assumption that it represents an objective property of populations. Members of different groups may perceive substantially different degrees of ideological polarization in the same society, an aspect that may either reinforce disagreement and conflict or offer potential avenues for resolution or de-escalation.

**Fig. 1.**
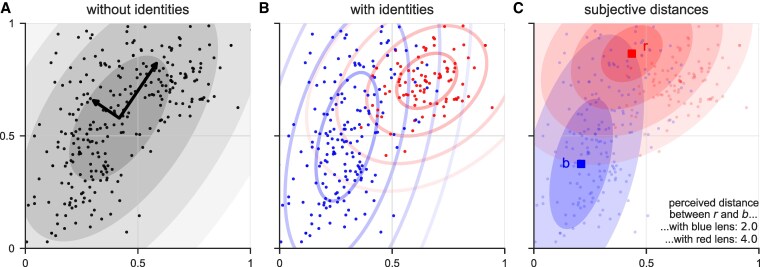
Illustrations of the model with fabricated opinion data. A) Opinions in the 2D space and the corresponding representation of the opinion space (arrows) used by individuals without identities. The ellipses indicate opinions that would be one, two, three, … unit distances from the center of this distribution. B) The same opinion data, but for individuals that affiliate with either a red or a blue identity group. There is a notable difference between the subjective representation of the opinion space—the lens—used by the red group, which is more homogeneous and, thus, characterized by a smaller variance, and the subjective representation used by the blue group, which exhibits a larger variance, especially along the issue represented on the *y*-axis. C) The distance between two opinions, *r* and *b*, is perceived asymmetrically by two individuals with different identities. The distance between *r* and *b* perceived by the individual holding opinion *r* (with red identity) is twice as large as the distance perceived by the individual holding opinion *b* (with blue identity). The method can be applied to continuous opinion data—typically in the context of opinion dynamics models—and discrete opinion data—typically obtained through surveys using Likert scales. To provide an intuitive and illustrative understanding of the method, we use here continuous data, but later we apply it to real-world, discrete empirical data.

We present here an analytical framework that integrates how modified perceptions are affected by social identity in the analysis of opinion data. Our method (see Materials and methods section for details) can analyze opinion data collected using standard approaches, in which individual opinions on specific issues such as “How much do you worry about climate change?” can be represented on a continuum between two extremes, 0 (“not at all”) and 1 (“extremely”). When individuals answer questions about many topics, we can then map their opinions on a shared multidimensional space (Fig. 1A), where each dimension represents a topic (for example, corresponding to a survey question). Disagreement can be measured objectively, from the perspective of a neutral observer, by calculating the Euclidean distance between the locations of two individuals in the opinion space. We focus here, however, on the fact that disagreement can be measured *subjectively* from the perspective of diverse individuals who affiliate with different social or political identities. In this case, opinions—and thus the level of disagreement and ideological polarization in the population—are seen through a group-specific, dynamic “lens.”

We hypothesize that the subjective representation of the opinion space—the lens (or viewpoint)—is determined by the principal components of the opinion distribution within the in-group. That is, the axes of the original, objective opinion space are rotated and scaled to match the principal components and their loadings. In a 1D opinion space, this procedure yields opinion distances that are normalized by the in-group standard deviation. For example, if members of an in-group unanimously agree on an issue (low variance in the in-group opinion distribution), the lens will amplify the distances they perceive in relation to other opinions. For multidimensional opinion spaces, the principal component approach not only scales each of the axes of the opinion space independently from each other, capturing the salience of the corresponding issue, but also allows for axis rotation, capturing the subjective perception of how opinion dimensions are related. For example, if a group considers opinions about environmental protection and climate change mitigation as interrelated, its members might not represent these opinions as two separate dimensions (environmentalism and climate change attitudes), but instead along a rotated set of axes—one representing the attitude towards mitigating adverse anthropogenic impacts in general, and the other representing a preference for climate change mitigation over environmental protection, or vice versa (the orthogonal axis).

Figure 1 provides a conceptual explanation of our model using fabricated 2D data in which individuals have either no social identity (Fig. 1A) or one of two different identities, leading to different perceptions (Fig. 1B). In the latter case, the blue group has strong internal agreement on the topic represented on the *x*-axis. Thus, individuals of this group “see” less variance on topic *X* than on topic *Y* and will consider individuals who deviate from their opinion in *x*-direction as much more discordant than individuals who deviate similarly in *y*-direction. Opinions perceived as equally distant from a point in the space thus lie on an ellipsis around that point rather than on a circle (as would be the case for an objective lens or for an isotonic lens, i.e. when the principal components have equal loadings). In Fig. 1A and B, we show concentric ellipses that correspond to one, two, … unit distances from the respective center of the distribution (in arbitrary units). The procedure reveals an asymmetry in how groups perceive differences between opinions. Figure 1C illustrates this with an example. The distance perceived between *r* and *b* is two units (two concentric blue ellipses) when seen with the blue lens and four units (four concentric red ellipses) when seen with the red lens. The asymmetric perception in this case implies that members of the relatively homogeneous red community will generally view members of the blue community as more divergent, while members of the blue community will view members of the red community as more coherent with their own beliefs.

Perceived polarization, *P*, represents an increase over time in the magnitude of disagreement in a society as perceived by its members. We define this disagreement as the average opinion distance that individuals measure between themselves and others. This measure can update in one of two ways, depending on how individuals adjust their lenses. First, an individual may not update the lens in response to changing opinion distributions. We refer to this as *pure* polarization, denoted $P=P_{1}$. Second, and in addition to this pure polarization, an individual may update the lens due to changes in how opinions vary in the in-group, which may either augment or reduce perceived differences. The perception of increased disagreement in this case may arise from pure polarization, $P_{1}$, as well as from changes in how the individual generally perceives opinions; we refer to this additional increase as *lens-specific* polarization, denoted $P_{2}$, and the perceived polarization yields $P=P_{1}+P_{2}$.

Our model allows us to disentangle the perception of polarization into components that may or may not be aligned. For example, when the opinion distribution contracts within a particular in-group (i.e. the opinion variance decreases), the group’s perception of the opinion landscape narrows such that opinion differences within the wider population will appear larger to its members. This narrowing induces lens-specific polarization, even though outsiders with fixed lenses might see the opinions in society as, on average, consistently distant (i.e. P>0 even if $P_{1}=0$). In general, it is not known how responsive people are to opinion changes within their in-groups, but we expect a time lag before individuals adjust their lenses in response to updated opinions within the in-group. To explore the possible extent to which lens-specific polarization matters, we consider a scenario in which individuals have full, up-to-date knowledge of opinions among their in-group and instantaneously adjust their lenses accordingly. We refer to the lens-specific polarization in this specific scenario as *instantaneous* lens-specific polarization, denoted as $P_{2}^{*}$. Table 1 provides a summary of the types of polarization considered.

**Table 1.** Definitions of polarization considered in this study.

Symbol	Name	Description
$P_{\mathrm{obj}}$	Objective polarization	Increase in disagreement, i.e. the average pairwise opinion distances in a fixed, objective opinion space. In empirical survey data, this opinion space is often given by a mapping of the Likert-scale response options, such as “Strongly disagree,” “Disagree,” …, “Strongly agree,” to (equally distant) numeric values, −2, −1, …, 2.
*P*	Perceived polarization	Perceived increase in disagreement as seen by individuals (average pairwise opinion distances through the respective subjective lenses); $P=P_{1}+P_{2}$.
$P_{1}$	Pure polarization	Perceived increase in disagreement due to changes in opinions (assuming fixed subjective lenses).
$P_{2}$	Lens-specific polarization	Perceived increase in disagreement when current opinions are viewed through the updated lenses vs. when they are viewed through the old lenses. When lenses are fixed, $P_{2}=0$.
$P_{2}^{*}$	Instantaneous lens-specific polarization	Lens-specific polarization under the assumption that individuals adjust their subjective lenses to the most up-to-date in-group opinion distribution. When accounting for delayed changes of lenses, estimates for $P_{2}$ range between 0 (fixed lenses) and $P_{2}^{*}$ (instantaneously updated lenses). In principle, however, $P_{2}$ is not limited by an upper bound because lenses used by individuals can be arbitrarily narrow or broad, whereas lenses in our model are tied to the variation of in-group opinions.

As we only consider ideological polarization and not other types of polarization, we omit the specification below.

**Fig. 2.**
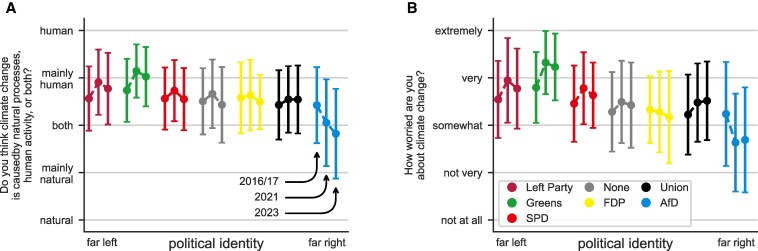
Climate change opinions over time by partisan identity. Average opinion (dots) and SD (error bars) of the participants’ responses to questions about the causes of climate change (A) and degree of concern about it (B) over the three survey waves. We grouped the participants by identity (i.e. the political party that they feel closest to), with nonpartisans grouped together as “None.”

We apply our model to empirical data collected by the European Social Survey (ESS) in 2016/2017 (wave 8), 2021 (wave 10), and 2023 (wave 11) (29–31). The data provide a representative distribution of opinions towards climate-related questions among German citizens. Moreover, participants in the survey also reported the political party they felt closest to and we use this as a proxy for the individuals’ political identities (see Materials and methods for details). This assumption is supported by extensive empirical evidence showing that partisan affiliation provides people with a reference frame and important cues to interpret the political world (25, 32, 33). Note that around half of the participants did not feel close to any party; we include these unaffiliated individuals as “nonpartisans” and, for convenience, assume that they consider all individuals as in-group regardless of their partisan identities. Figure 2 shows the average responses to the two climate-related questions we selected for our analysis and the standard deviation for each partisan group over the three survey waves.

Below, we will first illustrate how the choice of a subjective lens can distort the perception of disagreement in the German climate debate. We then apply the model to measure objective and perceived polarization, assuming subjective lenses. We show that these measures of polarization can change in nonnegligible ways if the subjective lenses of individuals are not fixed but adjust to changing in-group opinions. This effect may substantially shrink or amplify the perceived polarization and should therefore receive attention when quantifying or discussing societal polarization. The direction of the effect, however, is neither consistent nor predictable over time or across partisan groups. This indicates that members of different political groups may not even agree on whether social and political cleavages are becoming more profound or not.

## Results

**Fig. 3.**
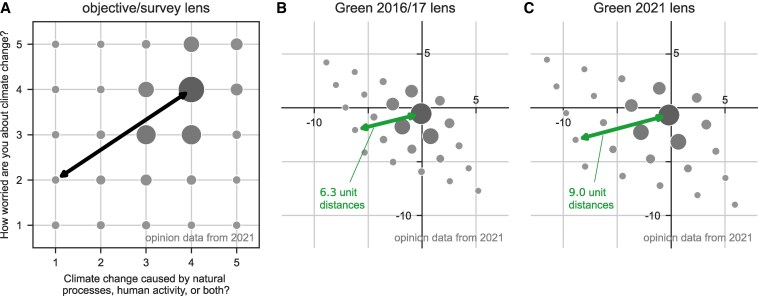
Distribution of climate-related opinions in 2021 seen from different viewpoints. The sizes of the gray circles indicate the relative frequencies of the answers. A) Opinion distribution shown on the objective 5×5 grid of possible responses to the two survey questions. Our model assumes that individuals perceive opinions in a subjective representation of that space, i.e. they view the opinion landscape through a lens shaped by the opinion variance within their in-group. The lenses that individuals considered in 2021 are, of course, not known and we assume that the individuals adjust their lenses over time. B and C) The same opinion distribution of 2021 (as in A), but viewed by an individual affiliating with the Green party and using the lens of 2016/2017 Greens, i.e. based on the opinion variance exhibited by the Greens in 2016/2017 (B), and by the same individual using the lens of 2021 Greens, i.e. based on the opinion variance exhibited by the Greens in 2021 (C). A Green using the “new” 2021 lens would perceive a substantially higher level of disagreement than the same Green using the “old” 2016/2017 lens (compare the green arrows in (B and C), which correspond to the perceived distances between the responses (4,4) and (1,2) in (A), seen with the two different lenses). This suggests a high degree of instantaneous lens-specific polarization $P_{2}^{*}$ for Green affiliates.

Figure 3A shows the climate change opinion data collected in 2021 (wave 10) from an objective viewpoint, depicting the distribution of responses as printed on the survey. The black arrow indicates the distance between two opinions ((4,4) and (1,2)). Figure 3B and C show how an individual affiliated to the “Greens” would see this distribution according to our model. If that individual used a lens based on 2016/2017 data (Fig. 3B), the distance between the two opinions (green arrows) would appear smaller than when that individual used a lens based on 2021 data (Fig. 3C). In general, the distribution of opinions perceived with the lens of 2021 is notably more spread out than the distribution perceived with the lens of 2016/2017, even though the opinion data are the same in all panels (Fig. 3). This happens because, between 2016/2017 and 2021, climate change opinions of the Greens homogenized and this manifests in a narrower 2021 lens. The narrower lens amplifies the opinion distances compared to the wider 2016/2017 lens. In other words, the mere updating from the old to the new lens leads Green affiliates to perceive an increase in disagreement, or—as we call it—lens-specific polarization.

**Fig. 4.**
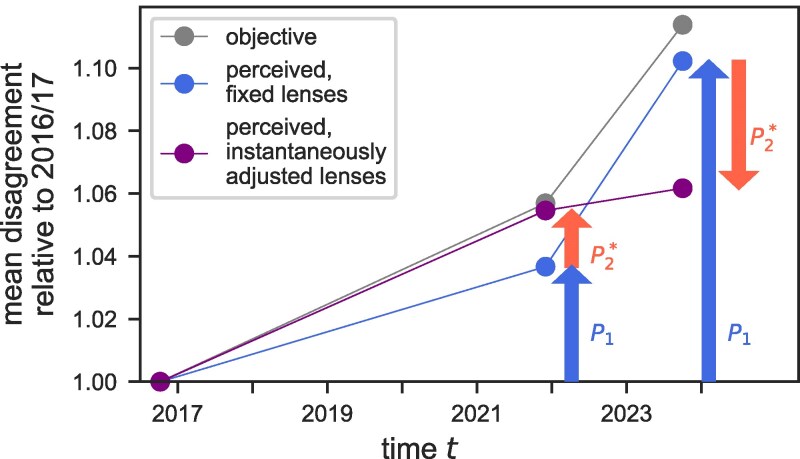
Effect of dynamic lenses on perceived disagreement about climate change among Germans. The mean disagreement (relative to 2016/2017) shown on the *y*-axis is calculated from objective pairwise opinion distances (gray dots) and from subjectively perceived opinion distances, assuming that individuals use fixed lenses calibrated to in-group opinions of 2016/2017 (blue dots) or that they use lenses adjusted instantaneously to the current in-group opinions (purple dots). The blue arrows indicate pure opinion polarization, $P_{1}$, which represents the increase in disagreement between 2016/2017 and 2021 and between 2016/2017 and 2023 perceived with fixed lenses. Perceived disagreement measured in this way has increased by roughly 10% (similar to the objective polarization). When individuals update their subjective lenses to the current in-group opinion distributions instantaneously, perceived polarization comprises pure opinion polarization, $P_{1}$ (blue arrows), and instantaneous, lens-specific polarization, $P_{2}^{*}$ (red arrows). The latter either further amplifies (2021) or shrinks (2023) the perceived polarization relative to opinions in 2016/2017.

By objective measures, German opinions on climate change have diverged between 2016/2017 and 2023. Specifically, we find that the average Euclidean distance between climate change opinions of the survey participants increased steadily over subsequent waves relative to 2016/2017 (Fig. 4, gray dots). Measuring polarization from the subjective viewpoints of the individuals but keeping their lenses fixed to the partisan opinion distributions of 2016/2017, we observe a similar increase in the mean disagreement over time, albeit slightly reduced in magnitude (Fig. 4, blue dots). However, this may not reflect the polarization that people really perceive if they adjust their lenses. Specifically, if perception relies on political identity similar to how we suggest here, and if people calibrate their lenses instantaneously to new in-group opinions, then polarization would have been perceived *larger* in 2021 and substantially *smaller* in 2023 (red arrows in Fig. 4) than if the lenses had remained fixed after 2016/2017. Consequently, on average, people would have perceived disagreement on climate change to remain nearly stable between 2021 and 2023 in this case (purple dots in Fig. 4). Taken together, the effects of lens-specific polarization may critically affect the level and variation of polarization perceived by individuals.

**Fig. 5.**
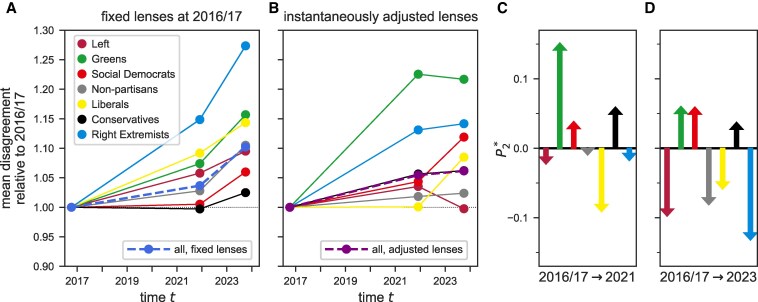
Effects of dynamic lenses on perceived disagreement about climate change across partisan groups in Germany. The mean disagreement perceived by each of the partisan identity groups relative to 2016/2017 is calculated assuming that individuals use fixed subjective lenses calibrated to in-group opinions of 2016/2017 (A) or that individuals instantaneously adjust those lenses to the current in-group opinions (B). For comparison, the dashed blue and purple lines repeat the (weighted) population average, as shown in Fig. 4. C and D) The difference between the scenarios assuming fixed or dynamic lenses, i.e. the difference between the dots in (B) and the dots in (A), is the instantaneous lens-specific opinion polarization, $P_{2}^{*}$. and is shown as arrows for each group separately in 2021 and 2023 (akin to the red arrows in Fig. 4).

The effect of (instantaneously) adjusting lenses on perceived polarization varies greatly across the different political groups (Fig. 5). For example, with dynamic lenses, the Greens perceive strong polarization between 2016/2017 and 2021—more than any other group and much more than expected from polarization with lenses fixed to in-group opinion distributions of 2016/2017 (Fig. 5A). At the same time, the Liberals (those affiliating with the party “FDP”) perceived almost no polarization during that time (Fig. 5B) even though their perceptions of polarization would be almost identical to those of the Greens if both groups used lenses fixed to their respective in-group opinions of 2016/2017 (Fig. 5A). There is some consistency in the effects of instantaneous lens-specific polarization within groups in 2021 and 2023—the arrows indicating $P_{2}^{*}$ point in the same direction for all groups in both time points (Fig. 5C and D)—, but the pattern is irregular, especially in relation to the magnitude of $P_{2}^{*}$. This indicates that the in-group distributions and, therefore, the group-specific lenses changed in notably different ways over the two time periods, likely reflecting differences in the ways the groups adapted to external and internal pressures.

## Discussion

We have presented a model to uncover the effects of partisanship on the way ideological polarization is perceived. The model formalizes how an individual’s subjective perception of the opinion landscape (which we call the lens) is tied to partisan identities and suggests how such perception adapts to actual changes of opinions. A homogenization (or heterogenization) of in-group opinions affects the way individuals evaluate differences in opinions within the broader population and causes them to perceive increased (or decreased) levels of ideological polarization. Using empirical data about opinions on climate change from German citizens, we showed that accounting for dynamic subjective perceptions importantly alters the polarization that people might experience and that this effect varies both across groups and over time.

Our model simultaneously accounts for two conceptions of polarization (4) that are rarely combined: polarization as issue alignment (or party sorting), which suggests that political cleavages between antagonistic groups extend to an increasing number of issues (21, 34, 35), and polarization as ideological divergence (36–38), which suggests that opinion differences between individuals objectively increase when measured by a neutral observer. Our measure captures both notions such that perceived polarization is not only determined by opinion heterogeneity within the overall population but is also shaped by the homogeneity of in-group opinions. This dual dependence is an important feature of any useful opinion polarization index, as proposed by Esteban and Ray (5) and many subsequent researchers (13, 15).

The novelty of our approach lies in the formalization of how an individual perceives the opinions of others and thus evaluates the degree of disagreement in a society through a group-specific lens. An important implication of our results is that polarization is a subjective, heterogeneous experience of individuals, rather than an objective societal feature. This is a departure from previous notions attributing differences in perceived polarization to erroneous perceptions and suggesting that metrics need to “correct” or de-bias the reported data to measure “true” polarization accurately (39). We consider the subjective, asymmetric and dynamic nature of perception as crucial, because in reality it is often this *perception* of ideological polarization by individuals that can undermine collective action (40, 41), can create a barrier to effective policy-making (42, 43), and can threaten social cohesion (1, 44).

The motivation behind our model of perception is closely related to the ideas underlying the “belief network” approach (10, 45–48). Belief networks describe relations or constraints between opinion dimensions, which are typically inferred from the correlations of opinions across individuals. For example, opinions on climate change mitigation and environmental protection are naturally related. Certain opinion dimensions (like religion or political identity (45)) can be central if they are highly related to (and, thus, predictive of) other opinion dimensions. With our approach focusing on opinion distances and perceived disagreement, we adopt a spatial rather than a relational understanding of belief systems (19) but we rely on a similar correlation- or variance-based inference method. In addition, we consider that belief systems (lenses in our model) are dynamic, adaptive, and heterogeneous across society—aspects that are widely under-explored in the belief network literature. Furthermore, we highlight the crucial influence of identity (partisan identity in our application) on belief systems and perceptions by formalizing how identities provide people with a frame of reference to evaluate the political world (32, 49–51). The inclusion of such heterogeneous, identity-driven and adaptive belief systems is important because there is evidence that belief systems of different political groups have indeed diverged in the past (20, 22). Consider, for example, the US debate on climate change: In the early 2000s, disagreement was common among both Democrat and Republican supporters. This changed around 2008, when climate change became a relevant political dimension with the election of Barack Obama, particularly for Democrats, who hold relatively homogeneous positions on the issue (8). Similarly, disagreement over abortion rights was common and tolerated among Republicans until the 1970s, before it became the passionate, identity-defining issue it is today (52). Our results suggest that such (asymmetric) changes in the belief systems of different groups can have a significant and nontrivial effect on the polarization people perceive.

The analytical framework we have presented remains hypothetical and would require empirical validation. While it seems plausible that individuals have a fairly good idea of the opinion distribution in their in-group (53, 54) and that their perceptions are shaped by this knowledge, we cannot be sure about how people actually adopt and operationalize viewpoints (or lenses) nor do we know the temporal scale over which their viewpoints may change. We generally expect the presence of a time lag between changes in opinions within the in-group and the adjustment of the viewpoint to such changes. Empirical studies could contribute to this by asking individuals to report how discrepant they perceive two contrasting political statements to be. People might not always agree on this, as our framework suggests. If this is the case, one could test whether asymmetries in perceptions are related to the degree of opinion variation the individuals observe within their social circles.

Empirical studies could also examine changes in perceived levels of disagreement over time and compare this to actual changes in opinion using longitudinal data (i.e. data collected from the same individuals repeatedly over time). In fact, many studies have shown that perceived disagreement often overshadows the objective divergence measured between opinions (14, 42, 55–58), including studies concerning misperceived attitudes towards climate change (40, 43). However, these studies generally measured misperceptions based on the participants’ perceptions of explicitly mentioned out-groups (“[As a Democrat], what do you think the typical Republican would think about issue X?”). In doing so, these studies conflated the subjective perception of opinions with other socio-psychological factors such as affective evaluation of out-groups (59), motivated reasoning (14, 25, 60, 61), “own-party cheerleading” (4), and anchoring biases (39, 56). We showed here that inflated perceptions of ideological polarization do not require drivers other than the assumption that perceiving opinions is a subjective process that is dynamically shaped by in-group variance. Factors, such as social segregation (62, 63), interrupted or biased flow of information (64, 65), cognitive biases (66), or affective polarization (67–69), might further amplify perceptions of polarization (70), but they are not necessarily required for the phenomenon to emerge.

It remains unclear what interventions could contribute to reducing polarization in a society. Lees and Cikara (57) have suggested that interventions could focus on correcting perceived beliefs rather than actual beliefs. This aligns with our findings but we add that focusing on the combined effects of perceptions and identities is critical. We see the value of our study in informing the debate around perceived ideological polarization rather than making policy recommendations (see the discussion in Ref. (71)), and our findings indicate potential avenues for research on interventions. How does amplifying diverse opinions within an in-group (e.g. by inviting two representatives per party to a TV debate instead of just a single one) affect the polarization the in-group members perceive in society? Also, how does emphasizing past disagreements within groups (e.g. by highlighting long-term opinion trends instead of just a snapshot obtained from a recent poll) affect these perceptions? Our findings suggest that increasing the awareness of in-group diversity or (past) disagreement could reduce perceptions of polarization by “widening the lenses” that group members use, but only if this intervention causes the individuals to update their lenses without diminishing the salience of their shared identity.

The value of our approach lies in highlighting the importance of heterogeneous and dynamic viewpoints for perceived ideological polarization. Even if a neutral observer perceives that the average level of disagreement in a society remains the same over time, members of that society could still experience ideological polarization if the opinions within their in-groups converge. Conversely, even if disagreement increases, people may not perceive this as polarization if the opinion variance within their in-groups follows the opinion variation between groups (i.e. across society). These effects may vary across individuals and over time. Our approach and findings may prove useful to data analysts seeking to quantify perceived polarization, to social scientists designing surveys for this purpose, and to anyone wondering about the discrepancy between the widespread, uncomfortable perception of living in a polarizing society and the reality that opinions are often more similar than people realize.

## Materials and methods

### Model to quantify perceived ideological polarization

In our model, the opinion of an individual *i* at time *t* is a numerical vector, $x_{i}(t)$, in the *m*-dimensional opinion space, which represents *m* topics of interest. We denote the set of opinion vectors of all *n* individuals as $X_{t}=\{x_{1}(t),x_{2}(t),\ldots x_{n}(t)\}$. In the following, we will often omit the time argument, *t*, for better readability. Ideological polarization generally measures some kind of differences between opinions. We denote the opinion distance, *d*, seen by an individual *i* between the own opinion and the opinion of another individual, *j*, at time *t* as $d(x_{i},x_{j}\;|\;L_{i})$, which is a function of the opinion vectors $x_{i}$ and $x_{j}$ and the subjective representation of the opinion landscape, $L_{i}$, used by the individual at time *t* (we call this a “lens,” more details in the following). We denote the set of lenses used by all *n* individuals at time *t* as $L_{t}=\{L_{1}(t),L_{2}(t),\ldots ,L_{n}(t)\}$. To quantify (overall) perceived disagreement in a society, we average the pairwise opinion distances perceived by each individual between themselves and all others:


(1) $$\bar{d}(t)\;:\;=\bar{d}(X_{t},L_{t})=\frac{1}{n}\cdot \frac{1}{n-1}\cdot \sum_{i=1\ldots n}\;\;\sum_{j=1\ldots n,j\neq i}\;d\left(x_{i},x_{j}\;|\;L_{i}\right).$$

Perceived ideological polarization, *P*, between times $t_{1}$ and $t_{2}$ is then


(2) $$P(t_{1},t_{2})=\bar{d}(t_{2})-\bar{d}(t_{1}),$$

where P>0 implies that individuals, on average, perceive a divergence of opinions and P<0 implies that they perceive a convergence of opinions. Note that here polarization refers to a dynamic process—the increase in disagreement (7, 72)—rather than to a property of a society at a given time. As we only consider ideological polarization in this manuscript and not other types of polarization, we drop the specification below.

In the most simple case every individual uses the same representation of the opinion space and $d(x_{i},x_{j})$ represents the Euclidean distance between vectors $x_{i}$ and $x_{j}$. However, perception of opinions is subjective and we define it according to the following two assumptions. First, individuals have fixed identities which separate them into distinct groups. Second, an individual *i* maps opinions in a subjective representation of the opinion space (or sees opinions through a subjective lens $L_{i}$), which is shaped dynamically to best represent the distribution of opinions within the in-group of that individual. We describe our formalization of these points below. For a less mathematical description of our method, we refer the reader to the illustrative example of perceived polarization between two individuals in SI Appendix, Section S1.

A subjective representation of the opinion space is characterized by its bases (e.g. the standard Cartesian space has the bases (1,0,0,…), (0,1,0,…), etc.) and this is in principle an arbitrary choice. We define that an individual *i* infers the bases of their subjective representation of the opinion space, $L_{i}$, from the principal components of the opinions among the in-group at a given time *t*, scaled by the variance that each principal component explains. Specifically, to calculate $L_{i}$, we first derive the covariance matrix $\mathrm{cov}(\{x_{k}\})$ of the opinions, $x_{k}$, at time *t* of all individuals *k* that are in the same identity group as individual *i*. From this, we obtain *m* rotated axes pointing in the direction of the eigenvectors $v_{1\ldots m}$ of the covariance matrix (the principal components) and scale each axis by the square root of the respective eigenvalue, $\sqrt{\lambda_{1\ldots m}}$, which represents the variance explained by the corresponding axis (or the loading of the principal components). This procedure yields a modified representation of the opinion space defined by *m* rotated and scaled axes, $\left\{\sqrt{\lambda_{k}}\cdot \vec{v_{k}}\right\}$, specific to each identity group and tied to the (in-group) opinion distribution $X_{t}$ at time *t*. Using these base vectors in the matrix $L_{i}$ individual *i* perceives the opinion *x* in coordinates, $x^{\prime }$, where


(3) $$\begin{array}{rl} x & =x_{1}^{\prime }\cdot {\left(\sqrt{\lambda_{1}}\cdot \vec{v_{1}}\right)}_{j}+x_{2}^{\prime }\cdot {\left(\sqrt{\lambda_{2}}\cdot \vec{v_{2}}\right)}_{j}+\ldots =\;:\;L_{i}\cdot x^{\prime }\\ & \;\;\;⟺\;x^{\prime }={\left(L_{i}\right)}^{-1}\cdot x. \end{array}$$

and the distance between opinion vector $x_{j}$ and opinion vector $x_{i}$ seen by the individual *i* yields:


(4) $$d(x_{i},x_{j}\;|\;L_{i}{)}^{2}=(x_{i}-x_{j}{)}^{T}\cdot {\left(L_{i}\right)}^{-T}{\left(L_{i}\right)}^{-1}\cdot (x_{i}-x_{j}).$$

The matrix $L_{i}$ can thus be understood as a group-specific “lens” (a subjective viewpoint) through which each individual sees distances between own opinions and the opinions of others. Note that $L_{i}=L=1$ yields the Euclidean distance between $x_{i}$ and $x_{j}$, representing the case of objective perception.

Let us first assume the case that individuals do not adjust their lenses between two times, $t_{1}$ and $t_{2}$, i.e. $L_{t_{1}}=L_{t_{2}}$, even if the opinion distributions within the groups change after $t_{1}$. Applying our definition of perceived polarization (Eq. 2), we obtain:


(5) $$\begin{array}{rl} P(t_{1},\;t_{2}) & =\bar{d}(X_{t_{2}},L_{t_{2}})-\bar{d}(X_{t_{1}},L_{t_{1}}{)}^{\text{fixed lenses}}\;\;\;\;\;\;\;\;\;\;\;\;\;\;\;\;\;\;\;\;\text{=}\\ & =\bar{d}(X_{t_{2}},L_{t_{1}})-\bar{d}(X_{t_{1}},L_{t_{1}})\;:\;=P_{1}(t_{1},t_{2}). \end{array}$$

We denote $P_{1}(t_{1},t_{2})$ as pure polarization.

In general, however, $L_{t}$ is not fixed. Individuals adjust their lenses over time to the evolving opinions and, thus, $L_{t_{1}}\neq L_{t_{2}}$. The degree to which people adjust their lenses to changes in the in-group opinion distribution is uncertain and there might be a time delay, *τ*, such that individuals at time *t* use lenses, $L_{t}$, which they inferred from an earlier opinion distribution $X_{t-\tau }$. Applying again our definition of perceived polarization (Eq. 2), we obtain:


$$\begin{array}{rl} P(t_{1},t_{2}) & =\bar{d}(X_{t_{2}},L_{t_{2}})-\bar{d}(X_{t_{1}},L_{t_{1}})=\left[\bar{d}(X_{t_{2}},L_{t_{2}})-\bar{d}(X_{t_{2}},L_{t_{1}})\right]+\left[\bar{d}(X_{t_{2}},L_{t_{1}})-\bar{d}(X_{t_{1}},L_{t_{1}})\right]=P_{2}(t_{1},t_{2})+P_{1}(t_{1},t_{2}). \end{array}$$

The perceived polarization in this case comprises pure polarization, $P_{1}$, and lens-specific polarization, $P_{2}$, where $P_{2}(t_{1},t_{2})=\bar{d}(X_{t_{2}},L_{t_{2}})-\bar{d}(X_{t_{2}},L_{t_{1}})$, represents the difference in perceived disagreement in the opinion distribution $X_{t_{2}}$ due to the updating of the lenses from $L_{t_{1}}$ to $L_{t_{2}}$.

To estimate the possible contribution of $P_{2}$ to the polarization perceived by the individuals, let us now assume that individuals are fully aware of the (updated) opinion distribution within their in-group—in other words, we assume a well-mixed in-group with respect to flow of information—and that they adjust their lenses to this distribution instantaneously (τ=0, denoted with an asterisk in the following). Thus, $L_{t_{2}}$ is inferred from the opinions $X_{t_{2}}$, and $L_{t_{1}}$ is inferred from $X_{t_{1}}$. We denote this as $L_{t_{2}}=L(X_{t_{2}})$ below. Then,


(7) $$\begin{array}{rl} P^{*}(t_{1},t_{2}) & =\bar{d}(X_{t_{2}},L_{t_{2}})-\bar{d}(X_{t_{1}},L_{t_{1}}){\;}^{\text{adjusted lens}}\;\;\;\;\;\;\;\;\;\;\;\;\;\;\;\;\;\;\;\;\;\;\text{=}\\ & =\bar{d}(X_{t_{2}},L(X_{t_{2}}))-\bar{d}(X_{t_{1}},L(X_{t_{1}}))\\ & =\left[\bar{d}(X_{t_{2}},L(X_{t_{2}}))-\bar{d}(X_{t_{2}},L(X_{t_{1}}))\right]\\ & \;+\left[\bar{d}(X_{t_{2}},L(X_{t_{1}}))-\bar{d}(X_{t_{1}},L(X_{t_{1}}))\right]\\ & =P_{2}^{*}(t_{1},t_{2})\;+\;P_{1}(t_{1},t_{2}). \end{array}$$

We denote $P_{2}^{*}(t_{1},t_{2})=\bar{d}(X_{t_{2}},L(X_{t_{2}}))-\bar{d}(X_{t_{2}},L(X_{t_{1}}))$ as instantaneous lens-specific polarization.

The Python code required to reproduce the results and figures in this manuscript is freely available in a public repository (https://doi.org/10.5281/zenodo.14617151) and can be easily adapted for use with new datasets.

### Opinion data on climate change

Our model of perceived ideological polarization can be applied to survey data that captures (i) the respondents’ opinions coded as ordinal values and (ii) social or political identities that play a relevant role in the topic. Here, we use the German subset of the European Social Survey (ESS) from wave 8 in 2016/2017 with n=2,852 (29), wave 10 in 2021 with n=8,725 (30)—conducted online due to COVID-19 restrictions—, and wave 11 in 2023 with n=2,420 (31). Focusing on climate change opinions, we selected two survey questions: *wrclmch* (“How worried are you about climate change?”) with response options on a five-point Likert-scale from 1 (“not at all worried”) to 5 (“extremely worried”) and *ccnthum* (“Do you think climate change is mainly natural/anthropogenic or both?”) from 1 (“entirely by natural processes”) to 5 (“entirely by human activity”). These two questions define a 2D, discrete opinion space $x_{i}\in \{1,2,3,4,5{\}}^{2}$. Assuming that the discrete response options, {1,2,…}, correspond to the respective values on a continuous scale, [0,1], we can apply our model to discrete data as well. From our analysis, we excluded respondents who did not give a valid answer to the questions, *ccnthum* and *wrclmch* (60, 277, and 14 in waves 8, 10, and 11), or who answered “climate is not changing” for *ccnthum* (3, 85, and 4). Responses to the two questions are highly correlated (Pearson correlation coefficient r=0.46).

We use affiliation with a political party as the most salient identity category for the German climate change debate. Specifically, we classified respondents according to which of the six most popular German parties they felt closest to: (i) “CDU/CSU,” the union of the conservative parties, (ii) “SPD,” the social democrat party, (iii) “Bündnis 90/Die Grünen,” the green party, (iv) “Die Linke,” the left party, (v) “FDP,” the economic liberal party, and (vi) “AfD,” the right-wing extremist party (*prtcl[e/f/g]de* in waves 8, 10, and 11). We excluded participants who affiliated with other parties (59, 177, and 41 in waves 8, 10, and 11) or refused an answer (55, 498, and 70). We classified those who felt no preference for any party or stated that they felt “not at all” close to their preferred party (*prtdgcl*) as nonpartisans with identity “None.” This nonpartisan identity group contained roughly half of the respondents (see SI Appendix, Table S1 for relative sizes of each identity group included in our analysis). We define that the lenses used by nonpartisans are derived in the same way as if they considered all participants as in-group members. Note that our analysis might under-represent the size of the right-wing extremist group, those affiliating with the “AfD.” While 7.9% of the eligible population voted for the “AfD” in the 2021 national election (10.3% of the votes with a turnout of 76.6%), their group represents only 2.2% of the 2021 ESS respondents (wave 10, see SI Appendix, Section S2). The final sample sizes used for the analysis were 2,681 in wave 8,7,807 in wave 10, and 2,295 in wave 11. To calculate representative averages over the sample population, we used the analysis weight recommended by the ESS (see SI Appendix, Section S3).

## Supplementary Material

pgaf184_Supplementary_Data

## Data Availability

The data underlying this article (Refs. (29–31)) are available through the ESS Data Portal at https://ess.sikt.no/.

## Preprints

A preprint of this article is published at https://doi.org/10.31235/osf.io/yau9s.
