# Supplementary material for: How opinion variation among in-groups can skew perceptions of ideological polarization
Source: PNAS Nexus. 2025 Jun 6;4(7):pgaf184. doi: 10.1093/pnasnexus/pgaf184 (PMC12218193; doi:10.1093/pnasnexus/pgaf184)
Supplement: pgaf184_Supplementary_Data [file pgaf184_supplementary_data.pdf]

SI Appendix to *How opinion variation among in-groups can skew perceptions of ideological polarization*

Peter Steiglechner[a,b,c,*], Paul E. Smaldino[d,e], Agostino Merico[a,b]

Friday 30th May, 2025

[a]Leibniz Centre for Tropical Marine Research (ZMT), Bremen, Germany
[b]Constructor University, Bremen, Germany
[c]Complexity Science Hub, Vienna, Austria
[d]University of California Merced, Merced, USA
[e]Santa Fe Institute, Santa Fe, USA
*To whom correspondence should be addressed: steiglechner@csh.ac.at

Supporting Information Text

S1 Simplified example of perceived polarisation

Imagine two individuals Alice and Bob. Alice holds a strongly supportive position on issue i and on issue j; that is, if we asked Alice about issues i and j in a survey with a 5-point Likert-scale, her response might be $x_A = \begin{pmatrix}5\\5\end{pmatrix}$. Consider now Bob, who would respond to these questions with $x_B = \begin{pmatrix}2\\1\end{pmatrix}$, being slightly dismissive of issue i and very dismissive of j. The objective distance between Alice's and Bob's opinions measured as the Euclidean distance would be $\sqrt{3^2+4^2} = 5$.

Imagine that Alice's in-group consists of five individuals with opinions $\begin{pmatrix}4\\4\end{pmatrix}$, $\begin{pmatrix}4\\4\end{pmatrix}$, $\begin{pmatrix}4\\5\end{pmatrix}$, $\begin{pmatrix}5\\4\end{pmatrix}$ and $\begin{pmatrix}5\\5\end{pmatrix}$. These individuals agree relatively strongly by holding very supportive positions on issues i and j. Bob's in-group also consists of five individuals with opinions $\begin{pmatrix}3\\1\end{pmatrix}$, $\begin{pmatrix}1\\2\end{pmatrix}$, $\begin{pmatrix}3\\3\end{pmatrix}$, $\begin{pmatrix}1\\4\end{pmatrix}$, and $\begin{pmatrix}3\\5\end{pmatrix}$, i.e., Bob's in-group members agree on being either neutral or very dismissive on issue i but they hold very diverse positions on issue j. Applying our method and assuming that Alice and Bob update their lenses instantaneoulsy to the current opinions in their respective in-groups, we find that Alice perceives the distance between Bob's opinions and her own as twice as large compared to how Bob perceives this opinion distance:

$$d\left(x_{\mathrm{Alice}}, x_{\mathrm{Bob}} | L_{\mathrm{Alice}}\right) = \sqrt{\begin{pmatrix}3 & 4\end{pmatrix} \cdot \begin{pmatrix}3.75 & -1.25\\ -1.25 & 3.75\end{pmatrix} \cdot \begin{pmatrix}3\\4\end{pmatrix}} \approx 8.0 \tag{1}$$

$$d\left(x_{\mathrm{Alice}}, x_{\mathrm{Bob}} | L_{\mathrm{Bob}}\right) = \sqrt{\begin{pmatrix}3 & 4\end{pmatrix} \cdot \begin{pmatrix}1.04 & -0.03\\ -0.03 & 0.38\end{pmatrix} \cdot \begin{pmatrix}3\\4\end{pmatrix}} \approx 3.8 \tag{2}$$

Note how Alices's lens is symmetric while Bob's lens, i.e, the matrix L_{Bob}, has smaller values in the bottom row. This implies that, viewed through Bob's lens, differences in the y-component of the opinion vector (relating to issue j) contribute less to perceived distance than differences in the x-component (relating to issue i). This reflects the larger variance in opinions on issue j within Bob's in-group.

Imagine that, over time, the opinions of Bob's in-group members converge on issue i and they now fully agree on a somewhat dismissive position (their udpated opinions are $\begin{pmatrix}2\\1\end{pmatrix}$, $\begin{pmatrix}2\\2\end{pmatrix}$, $\begin{pmatrix}2\\3\end{pmatrix}$, $\begin{pmatrix}2\\4\end{pmatrix}$, and $\begin{pmatrix}2\\5\end{pmatrix}$ with Bob's opinion remaining unchanged at $\begin{pmatrix}2\\1\end{pmatrix}$). According to our method, Bob now perceives any deviation from this narrow in-group consensus on issue i as very far (here, in fact, infinitely far) away from his own position, regardless of the opinion on the other issue j. Thus, Bob perceives the distance to Alice's opinion as strongly amplified. As a real-world example for this extreme scenario, imagine disagreeing with a person on preferring democracy over a dictatorship; you might perceive the political distance to that person as very large regardless of whether you agree or disagree on other issues such as migration or social security policies.

S2 Sampling weights

Equation 1 in the main article is a simplified version of the calculation of the perceived disagreement. Accounting for the sampling weights of the participants in the ESS, the disagreement yields:

$$\overline{d}(t) := \overline{d}(\mathcal{X}_t, \mathcal{L}_t) = \sum_i \frac{1}{\sum_{\mathrm{i=1..n}} w_i} \cdot \left(\sum_{\mathrm{j=1..n,\ j \neq i}} \frac{1}{\sum_i w_j - w_i} \cdot d\left(x_i, x_j \,|\, L_i\right) \right) \ . \tag{3}$$

where w_i are the analysis weights (column *anweight*) for each individual as provided by the ESS dataset. Note that we did not account for these weights when inferring the bases of the subjective representation of the opinion space of each group. The weights for each participant are in fact all very close to each other in the respective survey waves. Thus, disregarding these weights yields nearly exactly the same results.

S3 Representativeness of the data

Table S1: Shares of partisan identities in the ESS data included in our analysis for wave 8 (2016/17), wave 10 (2021), and wave 11 (2023) with or without those individuals that do not feel close to any party ('Non-partisan') and the overall size of the dataset in the column n after excluding invalid responses (see Materials and Methods).

Party	Left	Green	Social Democrat	Liberal	Conservative	Right-wing Extremist	Non-partisan identity	n
	Die Linke	Bündnis 90/Die Grünen	SPD	FDP	Union or CDU/ CSU	AfD	None	
2016/17	5.6 %	8.2 %	14.6 %	1.9 %	18.1 %	3.6 %	48.0 %	2681
... parties	10.8 %	15.7 %	28.1 %	3.7 %	34.7 %	7.0 %	—	
2021	4.0 %	12.2 %	12.4 %	5.1 %	12.7 %	2.2 %	51.5 %	7807
... parties	8.3 %	25.1 %	25.5 %	10.4 %	26.1 %	4.6 %	—	
2023	2.5 %	12.6 %	10.1 %	2.3 %	13.2 %	4.4 %	55.0 %	2295
... parties	5.5 %	28.0 %	22.4 %	5.1 %	29.3 %	9.7 %	—	

Table S1 shows the distribution of partisan identities as extracted according to the procedure described in the main article. To set this into perspective, we compare it with the voting shares that each party received in the German general election in September 2021, which occurred shortly before wave 10. The 2021 voter shares are 4.9 % for 'Die Linke', 14.8 % for 'Bündnis 90/Die Grünen', 25.7 % for 'SPD', 11.5 % for 'FDP', 24.1 % for 'Union' (or 'CDU/CSU'), and 10.3 % for 'AfD' (with a voter turnout of 76.6 %). Note that the ESS wave 10 questionnaire also asks its participants which party they voted for in the last national election, i.e. in September 2021 (*prtvfde2*): 5.5 % for 'Die Linke', 20.8 % for 'Bündnis 90/Die Grünen', 27.3 % for 'SPD', 13.3 % for 'FDP', 21.4 % for 'Union', and 6.6 % for 'AfD'. These results are similar to the question which party the respondents felt closest to (see Table S1). Comparing the national election results with the identities represented in the ESS sample, we conclude that the ESS sample seems to either under-represent the share of those identifying or voting for the 'AfD' or those voting for the 'AfD' do not state the 'AfD' as a party that they feel particularly close to. This likely introduces a bias in our analysis.
